# Identification of immunogenic proteins and generation of antibodies against *Salmonella* Typhimurium using phage display

**DOI:** 10.1186/1472-6750-12-29

**Published:** 2012-06-15

**Authors:** Torsten Meyer, Thomas Schirrmann, André Frenzel, Sebastian Miethe, Gerald F Gerlach, Katrin Strutzberg-Minder, Stefan Dübel, Michael Hust

**Affiliations:** 1 Technische Universität Braunschweig, Institut für Biochemie, Biotechnologie und Bioinformatik, Abteilung Biotechnologie, Spielmannstr.7, 38106 Braunschweig, Germany; 2 IVD GmbH Heisterbergallee 12, 30453 Hannover, Germany; 3 Present address: vaxxinova GmbH diagnostics, Johann-Krane-Weg 42, 48149 Münster, Germany

## Abstract

**Background:**

Solely in Europoe, *Salmonella* Typhimurium causes more than 100,000 infections per year. Improved detection of livestock colonised with *S.* Typhimurium is necessary to prevent foodborne diseases. Currently, commercially available ELISA assays are based on a mixture of O-antigens (LPS) or total cell lysate of *Salmonella* and are hampered by cross-reaction. The identification of novel immunogenic proteins would be useful to develop ELISA based diagnostic assays with a higher specificity.

**Results:**

A phage display library of the entire *Salmonella* Typhimurium genome was constructed and 47 immunogenic oligopeptides were identified using a pool of convalescent sera from pigs infected with *Salmonella* Typhimurium. The corresponding complete genes of seven of the identified oligopeptids were cloned. Five of them were produced in *E. coli*. The immunogenic character of these antigens was validated with sera from pigs infeced with *S.* Tyhimurium and control sera from non-infected animals. Finally, human antibody fragments (scFv) against these five antigens were selected using antibody phage display and characterised.

**Conclusion:**

In this work, we identified novel immunogenic proteins of *Salmonella* Typhimurium and generated antibody fragments against these antigens completely based on phage display. Five immunogenic proteins were validated using a panel of positive and negative sera for prospective applications in diagnostics of *Salmonela* Typhimurium.

## Background

*Salmonella spec*. is a genus of the *Enterobacteriaceae.* Two species are in the genus Salmonella: *S. bongeri* and *S. enterica*[1]*. Salmonella enterica* is classified in serogroups and serovars on the basis of their O- and H-antigens (somatic and flagellar antigens) [2,3]. So far, 2800 *Salmonella enterica* gene families and more than 2500 serovars are known. More than 1500 serovars belong to the subspecies *Salmonella enterica* subspecies *enterica*[4]. These pathogens cause foodborne gastrointestinal infections, usually through raw poultry and pork, but it can also be found in non-alcoholic beer or seafood. The subspecies *enterica* is the cause of 99% of human *Salmonella* infections. The prevailent serovars are Typhimurium and Enteritidis [4-7]. The most reported phage types for *Salmonella* Typhimurium are DT193, U302 and DT104. Infections with the latter two phage types increased in 2009 [5]. Human infections with phage type DT104 are particularly critical, because these strains are resistant to most of the commonly used antibiotics [6]. In Europe, *Salmonella* caused more than 130,000 reported infections in 2008 and 108,614 cases in 2009. In the US more than a million cases are estimated to occur [5,8].

Improved detection o f livestock colonised with *S.* Typhimurium would be very helpful to prevent foodborne diseases. In particular, infections in swine are difficult to diagnose, because the animals develop either no or only slight symptoms [9]. Only through continuous monitoring of the herds infections of humans can be prevented. Established methods for *S*. Typhimurium diagnostics are classically time-consuming, using microbiological cultures on different liquid and solid media [10,11], specific fluorescence labeled DNA probes [12], PCR [13] or recently, a quantum dot-based bead assay [14]. Currently, high throughput diagnostic of *S*. Typhimurium is performed by indirect ELISA [9,15,16]. The commercially available ELISA kits e. g. SALMOTYPE®- or Enterisol®-ELISA use a mixture of O-antigens of *Salmonella enterica* subspecies *entirca* serovars. They are based on the system established by Nielson et al. [15]. Because of this mixture, cross-reactions occur with other bacteria [15]. In addition, the sensitivity varies between the different ELISA assays [17]. For a sensitive and specific ELISA, immunogenic and species specific proteins are required [18]. The improvement of detection methods, as well as the development of new vaccines would be facilitated by the identification, characterisation and validation of previously unknown immunogenic proteins.

**Figure 1:**
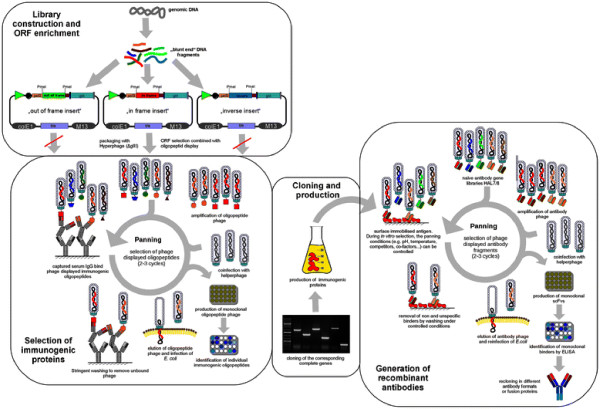
Schematic overview of the experimental pipeline allowing the selection and identification of immunogenic proteins, cloning and production of the immunogenic proteins and the generation of recombinant antibodies against these antigens.

The most common method for the identification of immunogenic proteins is 2D-PAGE of cultured bacterial pathogens and immunoblot using sera from infected patients or animals followed by mass spectrometry or microsequencing [19-24]. However, this method is limited. Differentially expressed proteins, e.g. dependent on pathogen-host interaction, can not be detected. Furthermore, weakly expressed antigens may also not be identified. In these cases, antigen phage display may circumvent these limitations. Our approach for the identification of immunogenic proteins is phage display. Phage display technology was invented by George P. Smith [25]. This methods can be used both for the selection of antibodies [26-29] and for the identification of immunogenic proteins from genomic or cDNA libraries [30-34]. Here, the cloning of randomly fragmented genomic DNA or cDNA into phage display vectors should allow, in theory, the display of all polypeptides encoded by the genome of the donor or all polypeptides encoded by the transcriptome of the donor, respectively [35,36]. In this study, we combined the identification of immunogenic proteins by M13 phage display using genomic libraries from *S.* Typhimurium with the selection of open reading frames without any subcloning steps (Figure 1 left part) in order to improve the library quality [37,38].

Afterwards, the genes corresponding to the identified immunogenic oligopeptides were cloned and produced in *E. coli* (Figure 1 middle part). Using our phage display based pipeline for the generation of human antibodies [39], we were able to generate human, recombinant antibodies against these antigens (Figure 1 right part).

## Results

### Generation of the Salmonella Typhimurium genomic phage display library

**Figure 2:**
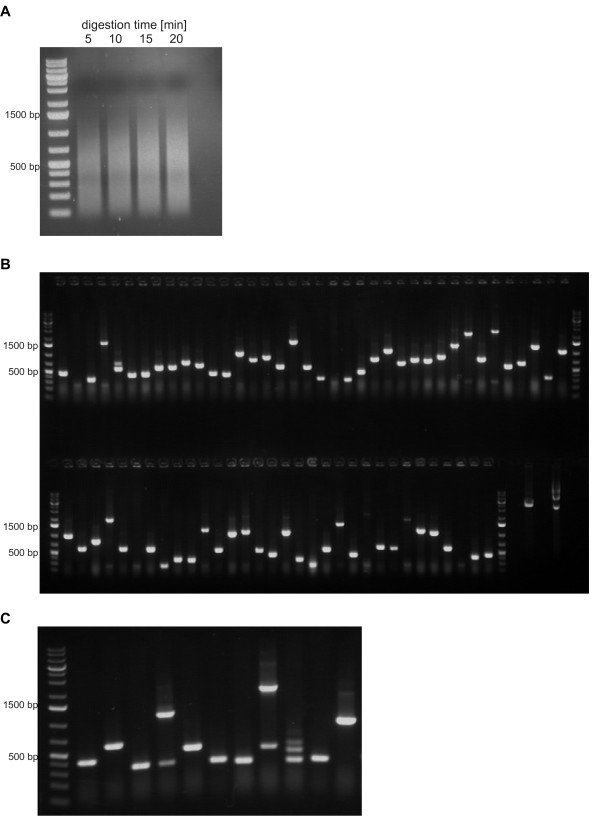
**A agarose gel of the digested genomic DNA of *Salmonella* Typhimurium.B** agarose gel of a colony PCR of the cloned genomic fragments. **C** agarose gel of a colony PCR of the inserts of Hyperphage packed phagemids.

Sonication of *Salmonella* DNA did not lead to clonable DNA fragments, whereas the sonication of *E. coli* DNA as a control could be cloned without problems (data not shown). Therefore, genomic DNA was digested with a mixture of the 4 base pair cutters *Dpn*I and *Alu*I and the 6 base pair cutter *Afe*I to construct the *Salmonella* Typhimurium genome library (Figure 2A). The digested DNA was cloned into pHORF3 [38] resulting in a library with 1.6x10^6^ independent clones. The insert rate and size was analysed by colony PCR (Figure 2B), which indicated that more than 90% of the clones contain an insert. The shortest inserts had a size of about 40 bp while the longest inserts had a lenght of 2500–3000 bp with an average length of about 500–800 bp. For the selection of open reading frames, the library was packaged with Hyperphage [37,40,41]. The packaged library was also analysed by colony PCR (Figure 2C) resulting in shorter DNA fragments compared to the initial library.

### Selection of immunogenic Salmonella Typhimurium oligopeptides

Pooled convalescent serum from pigs infected with *Salmonella* Typhimurium was used for the panning procedure. In total, two panning rounds were performed and 184 oligopeptide phage clones were analysed by ELISA for binding to serum IgGs. 58 oligopeptide phage clones with different gene fragments were found to bind to the porcine convalescent serum IgGs but not to the control serum IgGs. For 19 gene fragments, no homologous NCBI database hit was found. 31 of the identified proteins showed a higher similarity to other *Salmonellaentericia* serovars (Table 1). One of the selected gene fragments showed the best NCBI database hit to *Salmonella* Typhimurium, but the gene fragment was not in frame (Table 2). Seven oligopeptide phage clones with a good signal to noise ratio (three- to four-fold over background) in the ELISA and the highest homology to genes from *Salmonella enterica* spp. enterica serovar Typhimurium were selected for further characterisation (Table 3). Binding of the oligopeptide phage to convalescent serum IgGs, including the non-*Salmonella* Typhimurium hits and the out of frame *Salmonella* Typhimurium hit, was verified in an additional ELISA with piglet serum as negative control (data not shown).

**Table 1:** **Selected immunogenic* Salmonella *enterica proteins (without Salmonella serovar Typhimurium), including in frame and out of frame with gIII gene fragments**

***Protein***	***In frame with gIII***	***pHORF 3 insert size [bp]***	***NCBI reference sequence***	***Assigned Salmonella serovar***
ATP-dependent Clp protease ATP-binding subunit	yes	232	ZP_04657262	Tennessee
outer membrane ferrichrome receptor protein precursor	yes	58	YP_002635837	Paratyphi C
Rhs-family protein	yes	100	ZP_04657793	Tennessee
hypothetical protein SentesTyphi_03066	yes	294	ZP_03357516	Typhi
crotonobetaine/carnitine-CoA ligase	no	135	YP_002635709	Paratyphi C
transposase B	no	144	ZP_03224077	Kentucky
DNA polymerase I	no	348	ZP_03381877.	Typhi
nitrate reductase 2, gamma subunit	no	89	ZP_03366843	Typhi
putative phage terminase, large subunit	no	648	YP_002216031	Dublin
putative electron transfer flavoprotein alpha subunit	yes	58	YP_002636467	Paratyphi C
hypothetical protein	no	414	ZP_06535866	Typhi
Salmonellaentericaenterica_12643				
exonuclease V subunit gamma	no	86	ZP_04653610	Tennessee
bacteriophage Mu tail sheath protein	no	182	ZP_02663881	Schwarzen-grund
pts system, glucose-specific iibc component	no	109	ZP_02670079	Heidelberg
flagellar basal body P-ring protein	no	60	ZP_03371027	Typhi
hth-type tranScriptional regulator	no	710	ZP_02698922	Newport
DNA mismatch repair protein	yes	122	YP_002638485	Paratyphi C
colanic acid biosynthesis protein WcaK	no	270	ZP_02830210	Weltevreden
outer membrane fimbrial usher protein	yes	171	ZP_03336471	Typhi
uroporphyrinogen-III synthase	yes	49	ZP_03385917	Gallinarium
ATP-dependent metalloprotease	yes	130	ZP_03385917	Typhi
aminoglycoside/multidrug efflux system	yes	67	ZP_04657455.	Tennessee
penicillin-binding protein	no	56	ACN47454	Paratyphi C
C32 tRNA thiolase	yes	123	ZP_03365647	Typhi
hypothetical protein SG0660	no	324	YP_002225743.	Gallinarium
putative LysR-family transcriptional regulator	no	48	AET55395	Gallinarium
23 S rRNA methyluridine methyltransferase	yes	214	ZP_03356931	Typhi
hypothetical protein SeHA_C2934	yes	55	YP_002046718	Heidelberg
putative transport protein	yes	55	ACN44236.	Paratyphi C
hypothetical protein SPC_0823	yes	61	ZP_03365355	Typhi
nitrate reductase, alpha subunit	yes	298	ZP_03217505	Virchow

**Table 2:** **Selected immunogenic* Salmonella *Typhimurium proteins with out of frame gene with gIII fragments in pHORF3**

***Protein***	***pHORF3 insert size [bp]***	***NCBI reference sequence***
maltose ABC transporter periplasmic protein	260	NP_463094

**Table 3:** **Selected immunogenic* Salmonella *Typhimurium proteins**

***Protein***	***pHORF3 insert size [bp]***	***NCBI reference sequence***	***Molecular mass complete protein [kDa]***
putative dihydroxyacid dehydratase	118	NP_462432	62
putative electron transfer protein alpha	58	NP_459833	33
2,4-dienoyl-CoA-reductase	52	NP_462133	73
phage tail-like protein	70	NP_461635	23
putative dimethyl sulphoxide reductase	148	NP_460459	90
hypothetical protein STM14	58	ACY86745	7
putative carbohydrate kinase	40	CBG27384	72

### Cloning of the complete ORFs of the identified oligopeptides and antigen production

**Figure 3:**
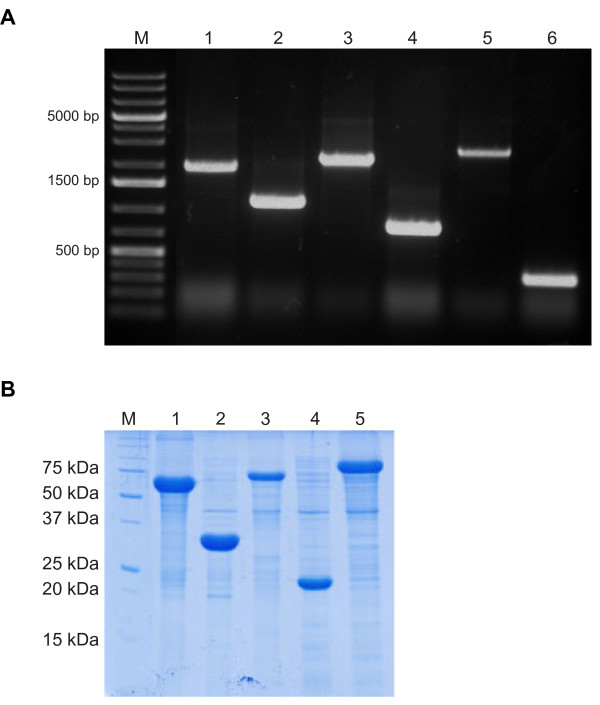
**A agarose gel of seven PCR amplified identified genes.** M: marker; 1: putative. dihydroyacid dehydratase; 2: putative electron transfer protein alpha; 3: 2,4-dienyl-CoA reductase, 4: phage tail like protein; 5: putative dimethyl sulphoxide reductase; 6: hypothecial protein STM14. **B** SDS-PAGE (12%) of five μL elution fraction of six IMAC purified immunogenic proteins. M: marker; 1: putative dihydroyacid dehydratase; 2: putative electron transfer protein alpha; 3: 2,4-dienyl-CoA reductase, 4: phage tail like protein; 5: putative dimethyl sulphoxide reductase.

The correspoding complete protein coding sequence of the identifed gene fragments was amplified from genomic DNA (Figure 3A) and cloned into pET21A+. When the ORF contained a leader peptide, it was replaced by the pelB leader peptide by cloning into pET21A + pelB. It was not possible to amplify the putative carbohydrate kinase encoding gene from the genomic DNA. The antigens were produced in 500 mL scale shake flasks, purified by IMAC and verified by SDS-PAGE (Figure 3B). Five of six antigens cloned into pET21A + were produced and purified successfully. It was not possible to produce the hypothetical protein STM14.

### Analysis of the identified immunogenic proteins with different pig sera

**Figure 4:**
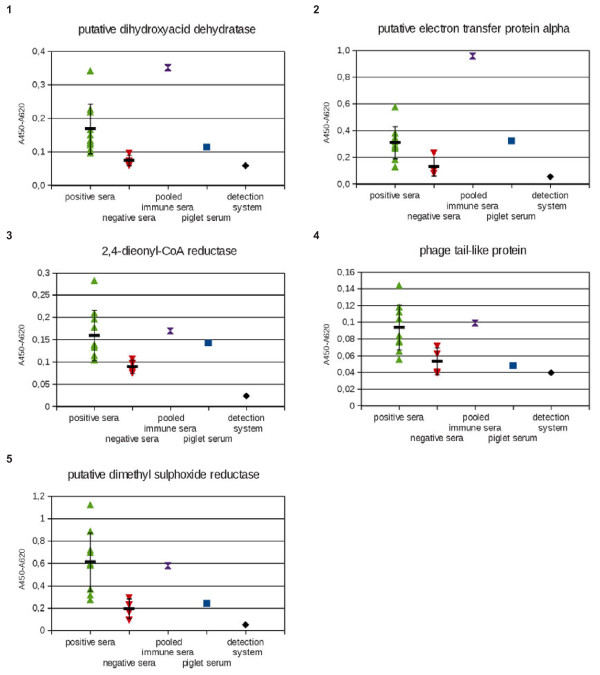
**ELISA for analysis of identified immunogenic* Salmonella *Typhimurium proteins with defined pig sera.1**: putative dihydroyacid dehydratase; **2**: putative electron transfer protein alpha; **3**: 2,4-dienyl-CoA reductase, **4**: phage tail like protein; **5**: putative dimethyl sulphoxide reductase. The sera from *Salmonella*-positive pigs (according to „Pig Screen“ ELISA) are marked dark blue, the sera from *Salmonella-*negative pigs (according to „Pig Screen“ ELISA) are marked orange, the mixture of positive sera used for the selection of immunogenic proteins is marked green, the piglet serum is marked dark red and the detection system control (only detection antibodies) is marked in light blue. For the panel of positive and negative sera arithmetic mean and standard deviation are given as black lines. 1 μg purified antigens were coated. The antigens were detected with diluted swine sera (1:200 in 2% MPBST) and goat anti-swine IgG HRP conjugate (1:10,000)

The five full size antigens were tested with sera from ten Salmonella Typhimurium positive classified pigs, using the commercial SALMOTYPE Pig Screen ELISA, and sera from four negative classified pigs. In addition, they were tested with the pooled immune positive sera used for the selection procedure and with a piglet serum as negative control (Figure 4).

9 out of 10 positive sera bound better to the antigen putative dihydroxyacid dehydratase compared to the negative sera. This means, that 9 of the 10 positive sera had a higher ELISA O.D. value compared to the negative serum with the highest ELISA O.D. value.

7 out of 10 positive sera bound better to the antigen putative electron transfer protein alpha compared to the negative sera. The pooled immune sera (positive control) contained either more or better binders against the antigens than any of the individual positive classified samples.

7 out of 10 positive sera bound better to the antigen 2,4-dieonyl-CoA-reductase compared to the negative sera. Here, the piglet serum revealed a high antigen binding capacity compared to the mixture of positive sera.

8 out of 10 as positive classifed sera bound better to the antigen phage tail-like protein compared to the negative classified sera.

8 out of 10 as positive classified sera bound better to the antigen putative dimethyl sulphoxide reductase compared to the negative classified sera.

Not all individual positive sera bound significantly better than all four individual negative sera. However, in general the positive sera showed better binding to all identified immunogenic proteins compared to the negative sera.

### Generation of recombinant human antibodies against the identified immunogenic proteins

Antibody fragments against all five antigens were selected using the human naive antibody gene library HAL7/8 [39]. Monoclonal binders were identified by antigen ELISA using soluble scFv fragments (data not shown). These binders were sequenced to identify unique binders and analysed using VBASE2 (http://www.vbase2.org) [42]. Human antibodies were successfully generated against all five antigens.

**Figure 5:**
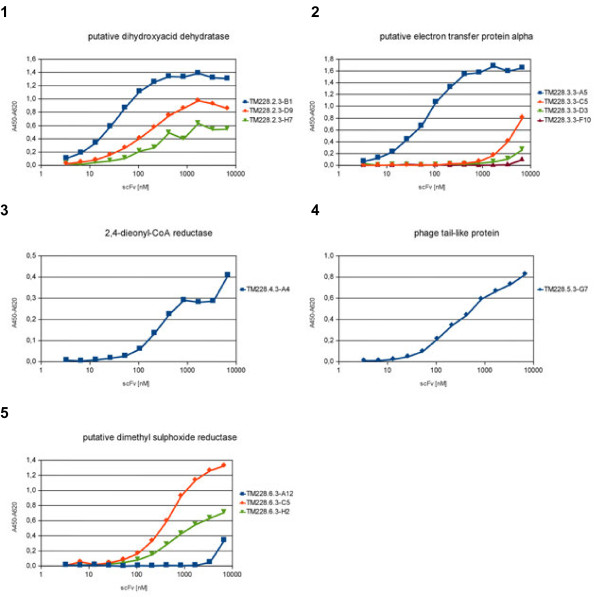
**Titration ELISA to analyse the selected anti-*Salmonella* Typhimurium antigens scFv.1**: putative dihydroyacid dehydratase; **2**: putative electron transfer protein alpha; **3**: 2,4-dienyl-CoA reductase, **4**: phage tail like protein; **5**: putative dimethyl sulphoxide reductase. 1 μg purified antigens were coated and detected with a dilution series of purifed scFv. Bound scFv fragments were detected with the anti myc 1-9E10 (1:500) and the goat-α-mouse-IgG (Fab spec.) HRP-conjugate (1:10,000).

The best binders were recloned into the pOPE101-XP vector [43], produced in 1.6 L scale in the LEX system and IMAC purified (Table 4). The yields were between 0.5 mg/L and 12 mg/L. Afterwards, the purified scFv were analysed by titration ELISA (Figure 5). The EC50 values of the scFv (monovalent) are given in Table 4. For four scFv it was not possible to determine the EC50, since the maximal binding was not reached in the titrations ELISA.

**Table 4:** **Characterisation of antibody fragments generated against* Salmonella *Typhimurium antigens. n.d. = EC50 not determined**

***scFv***	***Target***	***VH***	***VL***	***Yield [mg/L]***	***EC50 [nM]***	***EC 50 [μg/mL]***	***Immuno-blot***
TM228.2.3-B1	putative dihydroxyacid dehydratase	IGHV3-48*03	IGLV3-19*01	3.8	40	1.2	yes
TM228.2.3-D9	putative dihydroxyacid dehydratase	IGHV3-23*01	IGLV1-50*01	8.4	160	4.6	yes
TM228.2.3-H7	putative dihydroxyacid dehydratase	IGHV1-18*01	IGLV3-19*01	12.0	310	9.4	yes
TM228.3.3-A5	putative electron transfer protein alpha	IGHV1-46*01	IGLV7	4.3	78	2.3	yes
TM228.3.3-C5	putative electron transfer protein alpha	-	IGLV3-21*02	7.9	n.d.	n.d.	no
TM228.3.3-D3	putative electron transfer protein alpha	IGHV5-51*01	IGLV3-1*01	7.6	n.d.	n.d.	no
TM228.3.3-F10	putative electron transfer protein alpha	IGHV1-46*01	IGLV3-19*01	0.5	n.d.	n.d.	no
TM228.4.3-A4	2,4-dienoyl-CoA-reductase	IGHV3-15*01	IGLV3-19*01	1.3	310	9.4	no
TM228.5.3-G7	phage tail-like protein	IGHV1-2*02	IGLV1-47*01	7.4	310	9.4	yes
TM228.6.3-A12	putative dimethyl sulphoxide reductase	IGHV1-69*01	IGLV3-19*01	1.1	n.d.	n.d.	yes
TM228.6.3-C5	putative dimethyl sulphoxide reductase	IGHV4-31*03	IGLV1-44*01	1.5	625	18.8	yes
TM228.6.3-H2	putative dimethyl sulphoxide reductase	IGHV4-59*01	IGLV1-44*01	2.8	625	18.8	yes

The V genes are given according to VBASE2 (http://www.vbase2.org).

### Analysis scFv binding to Salmonella proteins by immunoblot

Binding to linear epitopes was analysed by SDS-PAGE of the antigens, followed by a Western Blot and an immunostain using the purified scFv. All binders to putative dihydroxyacid dehydratase, phage tail-like protein and putative dimethyl sulphoxide reductase bound linear epitopes. Three of the four binders to putative electron transfer protein alpha and the binder against 2,4-dienoyl-CoA-reductase did not bind in the immunoblot (Table 4).

## Discussion

Antibody phage display for generation of recombinant antibody fragments [39,44-47] and the identification of immunogenic proteins by phage display [30-32,38,48,49] are established methods. But in this work, for the first time a complete phage display based pipeline from antigen identification to the generation of the corresponding antibody fragments was shown. Oligopeptide phage display technology can expand the identification of immunogenic proteins compared to 2D-PAGE followed by mass spectrometry or microsequencing [32,38,48,50]. The identification of immunogenic proteins via oligopeptide phage display is independent of the natural expression rate of the immunogenic protein, which also allows the identification of low abundant proteins or proteins only produced in host-pathogen interactions. A disadvantage could be that only oligopeptides can be selected which can be secreted by the SEC pathway [38]. Interestingly, when using sonicated *S.* Typhimurium DNA, the transformation rates were in the range of 10^2^ - 10^4^ clones per transformation. This is very low compared to the transformation rates of 10^5^ for sonicated genomic DNA of *Mycoplasma hyopneumoniae* or 10^6^ clones for *E. coli*[38]. Hence, the sonication method appeared to be not applicable for some bacteria species or strains when constructing genomic libraries.

In this work, 58 different oligopeptides were bound by convalescent serum from pigs infected with *Salmonella* Typhimurium. Interestingly, many of the encoding gene fragments were not in frame with gIII and therefore, in theory, should not result in the production of functional oligopeptide-pIII fusion proteins. However, similar observations, that gene fragments encoding oligo- or polypeptides frequently contain frameshifts, have been described previously for selections by phage display [37,51]. For +1 frameshifts it is reported that oligo- or polypeptides are still displayed on phage particles with the same amino acid sequence as the corresponding constructs without a frameshift. One suggested explanation of this effect was the occurrence of RNA secondary structures. A second explanation could be the selection pressure against oligo- or polypeptides which are toxic for *E. coli* and thus may lead to a negative selection against these potential toxic proteins [52].

The most frequently identified oligopeptides did not show the best match with the *Salmonella* serovar Typhimurium (NCBI taxonomy IDs: 99287, 588858 and 568708), but instead with other *Salmonella* serovars. These antigens with a higher homology to other *Salmonella* serovars, could be interesting for further analyses. However in this work, we focused on the seven antigens with the highest homology to *Salmonella* Tyhphimurium. In contrast to former selections of immunogenic proteins using the pHORF system, where both new and known immunogenic proteins were selected [32,38], these seven antigens have not been described as immunogenic before. So far, five immunogenic proteins of *S.* Typhimurium were found using 2D-PAGE [53]. Putative dihydroxyacid dehydratase, putative dimethyl sulphoxide reductase and hypothetical protein STM14 of *Salmonella* Typhimurium have not been described as immunogenic before. The putative electron transfer protein alpha [54] is located on a pathogenicity island [55]. To date, 2,4-dienoyl-CoA-reductase of *S.* Typhimurium has not been identified as immunogenic, but interestingly, humans exhibiting anti-mitochondrial autoantibodies (AMA-positive), have also antibodies against the human 2,4-dienoyl-CoA-reductase [56]. For the phage tail-like protein, a bactericidal activity is described for some bacteria, e.g. *Pseudomonas*[57]. Immunogenic proteins from *S*. Typhimurium, which are used for diagnostics, are only rarely described in the literature. Described are OmpD [58] and a preparation of flagelates [59] for ELISA diagnostics. For nanobead based assays polyclonal antibodies against *Salmonella* were used whereupon the detailed antigens are unknown [14]. The V genes of the selected scFv against the five immunogenic proteins are mainly derived from the HV families 1 and 3 and from the LV families 1 and 3. Member of these gene families are preferentially selected from naive scFv libraries [60,61]. Only scFv with a lambda VL but no kappa VL were selected. Interestingly, also one VL domain only binder was selected. This is an artefact from library cloning since the insert rate of HAL7 is not 100% [39]. Functional VL domain dAbs have been described before [62].

The gold standard for diagnostics of *Salmonella* infections is microbiological culture [18]. Currently, for high throughput detection of *S*. Typhimurium, ELISA is the ideal method [9,15,16,58]. The commercially available ELISA kits use a mixture of O-antigens (LPS) or total cell lysate of *Salmonella enterica* subspecies *entirca* serovars. This mixture of antigens causes, cross-reactions with other bacteria [9,18]. A comparison of four different ELISA detection systems showed “both sample matrices, blood sera and meat juice, are suitable for antibody detection. However, the test sensitivity mainly depends on the respective cut-off used for the specific test” and “our findings indicate that the currently used LPS-ELISA systems have diagnostic uncertainties…” [9]. The use of one or a defined mixture of the selected immunogenic proteins and the corresponding antibody fragments will be useful to establish an ELISA based diagnostic kit with a higher specificity compared to the commercially available diagnostic kits.

## Methods

### Construction of the Salmonella Typhimurium genomic phage display library

*Salmonella* Typhimurium was cultivated in 2xTY medium [63] overnight at 34°C and 250 rpm. For isolation of genomic DNA, 6x 3 ml of the culture were used. The isolation was performed with the Quiaamp DNA Mini Kit according to the manufacturer’s instructions (Qiagen, Hilden, Germany). After purification, the DNA was digested for 35 min using three different blunt end-cutting restriction endonucleases (*Alu*I, *Afe*I, *Dpn*I) (NEB, Frankfurt, Germany). DNA fragments with a size up to 1200 bp, were used for cloning into the *Pme*I-restricted vector pHORF3 [38]. The ligated plasmids were transformed into *E. coli* Top10 F´ (Invitrogen, Karlsruhe, Germany) by electroporation.

### Enrichment of ORFs using Hyperphage

The enrichment of ORFs in the *S.* Typhimurium genomic library requires the display of the corresponding polypeptides on phage particles for the panning. Therefore, the library was packaged using Hyperphage [40,41] as described previously [37,38].

### Colony PCR

*E. coli* clones bearing pHORF3 were analysed by colony PCR using the primers MHLacZPro_f (5' GGCTCGTATGTTGTGTGG 3'), MHgIII_r (5' GGAAAGACGACAAAACTTTAG 3'), and the following protocol: 94°C 1 min, 56°C 0.5 min, 72°C 1.5 min, 25 cycles. The DNA was separated by 1% Agarose gel electrophoresis.

### Selection of immunogenic oligopeptides (Panning)

The panning was performed by following the protocol described before [38] with modifications. Six wells of a MaxiSorp® 96-well microtitre plate (MTP; Nunc, Wiesbaden, Germany) were coated with 150 μL 5 μg/mL goat anti-swine IgG in PBS [63] overnight. The wells were washed with phosphate buffered saline (PBS) supplemented with 0.1% Tween20 (PBST) (Roth, Karlsruhe, Germany). Afterwards, they were blocked with PBST supplemented with 2% (w/v) skim milk powder (2% MPBST) for 1 h. In parallel, several wells of a MaxiSorp® plate were coated with 150 μL 1 × 10^11^ cfu/mL Hyperphage in PBS overnight and blocked with 2% MPBST for 1 h. All washing steps were performed three times using PBST buffer and an enzyme-linked immunosorbent assay (ELISA) washer (Tecan Columbus, Crailsheim, Germany). A swine serum mixture (obtained from pigs after infection with *S.* Typhimurium and field sera) was diluted 1:10 in 2% MPBST and pre-incubated on MaxiSorp® MTP wells coated with Hyperphage for 1 h at RT, to remove serum IgG binding to the helperphage. After pre-incubation, the swine serum was incubated in goat anti-swine IgG-coated MTP wells for 2 h. After washing, 5 × 10^10^ cfu polypeptide phage particles of the Hyperphage-packaged *Salmonella* Typhimurium genomic library were incubated on the captured swine IgGs for 2 h. For the following panning rounds, 100 μL of amplified phage of the previous panning round were used. The non-binding polypeptide phage particles were removed by ten stringent washing steps. In the second and third panning round, the number of washing steps was increased to 20 and 30, respectively. Elution of bound phage particles was performed using 200 μL of 10 μg/ml trypsin (10 μg/mL trypsin in PBS) for 30 min at 37°C. Ten microlitres of the eluted phage solution were used for titration. Twenty millilitres of the *E. coli* TOP10 F´ cells were grown to an OD_600_ of 0.4 - 0.5 which were then infected with the remaining 190 μL of the eluted phage solution and incubated for 30 min at 37°C. Afterwards, the cells were harvested by centrifugation for 10 min at 3.220 × g. The bacterial pellet was resuspended in 250 μL 2 × TY medium (1.6% [w/v] tryptone, 1% [w/v] yeast, 0.5%[w/v] NaCl) containing 100 mM glucose and 100 μg/mL ampicillin (2 × TY-GA), plated onto 15 cm 2 × TY-GA agar plates and incubated overnight at 37°C. Grown colonies were harvested in 5 mL 2 × TY-GA medium using a Drigalsky spatula. Fifty millilitres of 2 × TY-GA medium were inoculated with 200 μL bacteria culture and grown to an OD_600_ of 0.4-0.5 at 37°C and 250 rpm in a shaking incubator. Five millilitres of bacterial culture corresponding to about ~2.5 × 10^9^ cells were infected with 5 × 10^10^ cfu Hyperphage, incubated at 37°C for 30 min without shaking and another 30 min with shaking at 250 rpm. The infected cells were harvested by centrifugation for 10 min at 3.220xg. The pellet was resuspended in 30 mL 2xTY medium containing 100 μg/mL ampicillin and 50 μg/mL kanamycin (2 × TY-AK). Phage particles were produced at 30°C and 250 rpm overnight. On the following day, the supernatant containing phage particles was collected.

### Production of individual oligopeptide phage clones for screening

For phage production, polypropylene 96-well U bottom plates (Greiner bio-one, Frickenhausen, Germany) containing 175 μL 2xTY-GA per well were inoculated with single *E. coli* colonies from the phage titration plates of the third panning round and incubated at 37°C with constant shaking at 850 rpm (thermo shaker PST60-HL4, lab4you, Berlin, Germany) overnight. A new plate with 165 μL 2xTY-GA per well was inoculated with 10 μL of the overnight cultures and incubated at 37°C and 850 rpm for 2 h. Afterwards, the bacteria were infected with 5x10^9^ cfu Hyperphage/well and incubated at 37°C without shaking for 30 min, followed by shaking at 850 rpm for 30 min. The MTP plate was centrifuged at 3,220xg for 10 min and the supernatants were discarded. Afterwards, the bacterial pellets were resuspended in 175 μL/well 2xTY containing 100 mg/mL ampicillin and 30 μg/mL kanamycin (2xTY-AK) and incubated at 30°C at 850 rpm overnight for phage production. The bacteria were pelleted again and the supernatants were transferred to a new plate. The phage were precipitated with 1/5 volume 20% PEG/2.5 M NaCl solution at 4°C for 1 h and centrifuged at 3,220xg for 1 h. The phage pellet was dissolved in 150 μL PBS and residual bacteria were removed by another centrifugation at 3,220xg for 5 min. The phage containing supernatants were stored at 4°C or directly used for ELISA.

### Screening of individual oligopeptide phage clones

For phage ELISA, the produced polypeptide phage particles were captured. Here, 100 μL of 250 ng/mL mouse anti-M13 (B62-FE2, Progen, Freiburg, Germany) in PBS were coated at 4°C overnight. After coating the wells were blocked with 2% MPBST. Between each incubation step the wells were washed three-times with PBST using an ELISA washer. 150 μL of the monoclonal phage production were incubated for 2 h. The pig convalescent serum was diluted 1:200 in 2% MPBST supplemented with 1/10 volume *E. coli* cell lysate and 1x10^10^ cfu Hyperphage/mL, added to the captured phage particles and incubated for 2 h. The bound pig IgGs were detected with goat anti-swine IgG conjugated with horseradish peroxidase (HRP) (1:1000) for 1.5 h and visualised with TMB (3,3´,5,5´-tetramethylbenzidine) substrate. The staining reaction was stopped by adding 100 μL 1 N sulphuric acid. The absorbances at 450 nm and scattered light at 620 nm were measured and the 620 nm value was subtracted using a SUNRISE microtiter plate reader (Tecan, Crailsheim, Germany).

### Cloning of the fullsize gene fragments

The corresponding proteins of the identified immunogenic polypeptides were amplified by PCR using the genomic DNA and the following oligonucleotide primers: TM208-forward (5´ AAGGAGATATACATATGAGCCAAAAATGTCAACATGCT 3´), TM208-reverse (5´ CTCGAGTGCGGCCGCTTTAAGCCAGGCTCCGGCCATTAA 3`) for putative dihydroxyaciddehydratase, TM209-forward (5´ AAGGAGATATACATATGGCTTCTTTAGTTATTGCTGAACAT 3´), TM209-reverse (5´ CTCGAGTGCGGCCGCTAATTTATCGATAAGTTCAGGTAC 3´) for putative electron transfer protein alpha, TM210-forward (5´ AAGGAGATATACATATGAGCTACCCGTCGCTGTTCGCCCCG 3´), TM210-reverse (5´ CTCGAGTGCGGCCGCAATCTCCAGTGCCAGTCGGGTGCC 3´) for 2,4-dieonyl-CoAreductase, TM211-forward (5´ AAGGAGATATACATATGAATAGTCTGTTGCCGCCGGGTTCG3´), TM211-reverse (5´ CTCGAGTGCGGCCGCTGGATTCACTCTCATTGTGTCAAT 3´) for phage tail-like protein, TM212-forward (5´ CAGCCGGCCATGGCTTCAATGAATAAAGCAGTCAGTAGTGAG 3´), TM212-reverse (5´ CTCGAGTGCGGCCGCACGTGCCGGGCGGTATTCGCGCCA 3´) for putative carbohydratekinase, TM213-forward (5´ CAGCCGGCCATGGCTGCGGTGCAGCAGGCTATGCGCAACGAA 3´), TM213-reverse (5´ TGCGGCCGCAAGCTTGATTTTTTCGATTTCCACCAGATTTGT 3´) for putative dimethyl sulphoxide reductase chain A1, and TM214-forward (5´ AAGGAGATATACATATGATGTCAGCATGTTTTTTTGGCCGA 3´), TM214-reverse (5´ CTCGAGTGCGGCCGCATCAATTATTTTGGTGAGTGTTTG 3´) for hypothetical protein STM14. The PCR was performed using the following protocol: 98°C 30 sec, 98°C 10 sec, 60°C 20 sec, 72°C 120 sec, 24 cycles. The DNA was separated by electrophoresis in a 1% agarose gel. The bands of interest were cut out from the gel, the DNA was eluted and used for cloning into pET21A + or pET21A + pelB. After ligation the plasmids were transformed into *E. coli* BLR-DE3. Positive clones were identified by using colony PCR using the oligonucleotide primers MHpET21_f1 (5´ GAGCGGATAACAATTCCCC 3´) and MHpET21_r1 (5´ GCAGCCAACTCAGCTTCC 3´).

### Production of the immunogenic proteins

Five hundred mL 2xTY-GA medium were inoculated with 5 mL overnight culture and cultivated to an O.D._600_ of 1.0 at 37°C and 250 rpm. The expression was induced with 1 mM IPTG (final concentration) overnight. Cells were harvested by centrifugation at 7.500 xg for 15 min. Lysis was performed with 1 mg/mL lysozyme and 5 μg/mL DNAseI in 15 mL His-tag binding buffer pH7.4 (20 mM Na_2_HPO_4_, 0.5 M NaCl, 10 mM Imidazol). For Isolation of inclusion bodies 8 M Urea was added. The purification was performed under denaturing conditions with FastFlow Sepharose (GE Healthcare) loaded with nickel. The Sepharose was washed with 10 mM, 30 mM and 60 mM imidazole (20 mM Na_2_HPO_4_, 0.5 M NaCl, 10, 30 or 60 mM Imidazol). For elution, 5 mL 100 mM EDTA in PBS supplemented with 8 M urea were used.

### SDS-PAGE

Antigens were analysed by 12% SDS-PAGE using a Protean II Minigel system (BioRad Inc, München, Germany) according to [63]. Protein gels were stained with coomassie blue.

### Enzyme linked immunosorbent assay (ELISA) for verification of immunogenic proteins

One μg of antigen was coated to 96 well microtitre plates (MaxiSorp, Nunc) in 50 mM NaHCO_3_ pH 9.6 overnight at 4°C. After coating, the wells were washed three times with PBST and blocked with 2% MPBST for 1.5 h at RT, followed by three washing steps with PBST. For serum ELISA, sera were diluted 1:200 in 100 μL 2% MPBST and incubated in the antigen coated plates for 1.5 h at RT, followed by three PBST washing cycles. Bound pig IgGs were detected with goat anti-swine IgG HRP conjugate (1:10,000) (Dianova, Hamburg, Germany). The visualisation was performed with TMB (3,3´,5,5´-tetramethylbenzidine) as a substrate and the staining reaction was stopped by adding 100 μl 1 N sulphuric acid. Absorbance at 450 nm was measured by using a SUNRISE™ microtitre plate reader (Tecan, Crailsheim, Germany).

### Generation of antibodies against the identified antigens

The selection of recombinant antibodies was performed according to [64] with modificiations. In short, pannings were performed in 96 well microtitre plates (MaxiSorp, Nunc, Wiesbaden, Germany). One μg of antigen was coated in PBS pH 7.4 [63] overnight at 4°C. The antigen-coated wells and wells for the preincubation of the library were blocked with 2% MPBST. In each case 2.5x10^11^ phage particles of the human naive antibody gene libraries HAL7 and HAL8 [39] were diluted in PBST with 1% skim milk and 1% bovine serum albumin (BSA) and preincubated for 1 h. The supernatant, containing the depleted library, was incubated in the antigen-coated wells at RT for 2 h followed by 10 washing steps with PBST. Afterwards, bound scFv phage particles were eluted with 200 μL trypsin solution (10 μg/mL trypsin in PBS) at 37°C for 30 min. The supernatant containing eluted scFv phage particles was transferred into a new tube. Ten μL of eluted scFv phage were used for titration as described before [64]. Twenty mL *E. coli* XL1-Blue MRF' (Agilent, Böblingen, Germany) culture in the logarithmic growth phase (O.D._600_ = 0.4 - 0.5) were infected with the remaining scFv-phage at 37°C for 30 min without shaking. The infected cells were harvested by centrifugation for 10 min at 3220xg and the pellet was resuspended in 250 μL 2xTY medium [63] supplemented with 100 mM glucose and 100 μg/mL ampicillin (2xTY-GA), plated on a 15 cm 2xTY agar plate supplemented with 100 mM glucose and 100 μg/mL ampicillin and incubated overnight at 37°C. Grown colonies were harvested with 5 mL 2xTY-GA. Thirty mL of 2xTY-GA were inoculated with 100 μL of the harvested colony suspension and grown to an O.D._600_ of 0.4 to 0.5 at 37°C and 250 rpm. Five mL bacteria suspension (~2,5x10^9^ bacteria) were infected with 5x10^10^ helperphage M13K07 (Stratagene), incubated at 37°C for 30 min without shaking, followed by 30 min at 250 rpm. Infected cells were harvested by centrifugation for 10 min at 3220 xg and the pellet was resuspended in 30 mL 2xTY supplemented with 100 μg/mL ampicillin and 50 μg/mL kanamycin (2xTY-AK). Antibody phage were produced at 30°C and 250 rpm for 16 h. Cells were harvested by centrifugation for 10 min at 3220xg. The supernatant containing the antibody phage (~1x10^12^ cfu/mL) were directly used for the next panning round or stored at 4°C for a few days.

### Production of scFv in microtitre plates (MTPs)

The identification of monoclonal binders was performed as described before [43]. In brief, 96-well MTPs with polypropylene (PP) wells (U96 PP 0.5 mL, Greiner, Frickenhausen, Germany) containing 150 μL phosphate buffered 2xTY-GA [63] (2xTY-GA supplemented with 10% (v/v) potassium phosphate buffer (0.17 M KH_2_PO_4_, 0.72 M K_2_HPO_4_)) were inoculated with colonies from the titration plate of the third panning round. MTPs were incubated overnight at 37°C at 1000 rpm in a MTP shaker (Thermoshaker PST-60HL-4, Lab4You, Berlin, Germany). A volume of 180 μL phosphate-buffered 2xTY-GA in PP-MTP well was inoculated with 10 μL of the overnight culture and grown at 37°C and 800 rpm for 2 h. Bacteria were harvested by centrifugation for 10 min at 3220xg and 180 μL supernatant were removed. The pellets were resuspended in 180 μL buffered 2xTY supplemented with 100 μg/mL ampicillin, 100 mM sucrose an 50 μM isopropyl-beta D thiogalacto pyranoside (IPTG) and incubated at 30°C and 800 rpm overnight. Bacteria were pelleted by centrifugation for 10 min at 3,220 xg and 4°C. The scFv-containing supernatant was transferred to a new PP-MTP and stored at 4°C before analysis.

### Identification of monoclonal scFv using ELISA

Antigen coating was performed as described above (Enzyme linked immunosorbent assay (ELISA) for verification of immunogenic proteins). For identification of binders, supernatants containing monoclonal scFv were incubated in the antigen coated plates for 1.5 h at RT followed by three PBST washing cycles. Bound scFv were detected using murine mAb 9E10 which recognises the C-terminal c-myc tag and a goat anti-mouse serum conjugated with horseradish peroxidase (HRP) (Sigma; 1:10,000).

The detection was performed as described above.

### Production of scFv in the LEX system

The large-scale expression system (LEX) (Harbinger Biotech, Toronto, Canada) was used for production of scFv. *E. coli* (XL1-Blue-MRF') was cultivated in 2 L glass bottles up to a cultivation volume of 1.5 L. To obtain sufficient oxygenation and mixing of the culture, the bottles were connected to an air manifold, which allows a general air flow rate of 4–6 L/min. A thermostat-controlled water bath was used for regulating the temperature of the cultivation. 50 ml TB supplemented with 100 μg/mL ampicillin were inoculated with a glycerol stock of each scFv clone and the culture was grown over night at 37°C. Glass bottles with 1.5 L TB supplemented with 100 μg/mL ampicillin and 500 μL antifoam 204 (Sigma, München, Germany) were inoculated with the overnight culture. The O.D._600_ was adjusted to 0.1 and incubated at 37°C until an O.D._600_ of 1.5 to 5 was reached. The temperature of the water bath was then reduced to 25°C. After 1 h, scFv expression was induced by addition of 50 μM IPTG. The cultivation was continued for 3 h resulting in a final O.D._600_ of 2 to 7 depending on the antibody clone. *E. coli* cells were harvested by centrifugation at 4,400xg (Sorvall Zentrifuge RC6 Plus, Rotor F9S-4x1000Y) for 10 min at 4°C. The pellet was resuspended in 60 mL ice-cold PE-buffer pH 8 (20% (w/v) sucrose, 50 mM Tris, 1 mM EDTA) and was incubated on ice for 20 min while shaking. Afterwards the sample was centrifuged at 20,000xg and 4°C for 30 min (Sorvall Zentrifuge RC6 Plus, Rotor F12-6x500y). The supernatant (periplasmatic preparation) was filled into a fresh glass bottle and kept on ice. The pellet was re-suspended in 60 mL ice-cold OS-buffer (5 mM MgSO_4_ in dH_2_O) and was incubated on ice while shaking. After 20 min the preparation was centrifuged at 20,000xg and 4°C for 30 min (Sorvall Zentrifuge RC6 Plus, Rotor F12-6x500y). The supernatant (osmotic shock fraction) was combined with the periplasmatic preparation and was used for protein purification.

### IMAC purification of scFv

Antibody fragments were purified by affinity chromatography using IMAC. Chromatography using Profinia (BioRad) and 1 mL FF-crude column (GE Healthcare, München, Germany) was performed according to the manufacturer's instruction. The protein solution was adjusted to 10 mM imidazol containing buffer (20 mM Na_2_HPO_4_, 500 mM NaCl, 10 mM imidazol) for loading. The column was washed one time with 10 mM imidazol buffer (20 mM Na_2_HPO_4_, 500 mM NaCl, 10 mM imidazol). Five hundred mM imidazol was used for elution, followed by desalting and storage in PBS.

### Titration ELISA using scFv

For the scFv titration ELISA the antigen was coated as described above (Enzyme linked immunosorbent assay (ELISA) for verification of immunogenic proteins). The ELISA was performed as described above (Identification of monoclonal scFv using ELISA) with one modification: a dilution series of IMAC purified scFv was used instead of the scFv supernatant. The EC50 values (antibody concentration at the half maximal binding) are deduces from this titration ELISA.

### Detection of the immunogenic proteins by immunostain using scFv

Purified immunogenic proteins were separated by 12% SDS-PAGE. Western Blotting on PVDF (Polyvinylidenfluorid) membranes of gels was performed using the Mini Trans-Blot® system (BioRad). The membrane was blocked with 2% (w/v) skimmed milk powder in PBST over night.

The antigens were detected with 20 μg/mL scFv for 1 h at RT. The scFv myc-tag was detected with mouse anti myc-tag (9E10, Sigma, Taufkirchen, Germany) for 1 h, followed by goat anti-mouse (Fc specific) (Sigma) conjugated with alkaline phosphatase (1:20,000) for 1 h. The visualisation was performed by addition of BCIP (5-bromo-4-chloro-3-indolyl phosphate) and NBT (nitroblue tetrazolium).

## Conclusion

A “pipeline” from antigen identification to the generation of recombinant antibodies using phage diplay was shown. Here, novel immunogenic proteins of *Salmonella* Typhimurium were identified using phage display and validated using a panel of positive and negative sera. Afterwards, recombinant human antibody fragments were generated against these marker proteins.

## Competing interests

The authors declare that they have no competing interests.

## Authors’ contributions

TM and SM performed most of the experiments and helped to draft the manuscript. TS and AF performed some of the experiments and participated in the design and coordination of the study. JSS, SD, GFG and KSM particpated in the design and coordination of the study and helped to write the paper. MH and JSS wrote the grant application. MH particpated in the design and coordination of the study wrote the publication. All authors read and approved the final manuscript.
